# Supplementary material for: Mapping the evolution of fertility support policies in China: A content and instrumental analysis
Source: PLoS One. 2025 Oct 9;20(10):e0332137. doi: 10.1371/journal.pone.0332137 (PMC12510515; doi:10.1371/journal.pone.0332137)
Supplement: S1 Appendix — (ZIP) [file pone.0332137.s001.zip › S1 Appendix. 226 original policy documents/100-企业职工带薪年休假实施办法(FBM-CLI-4-108957).doc]

企业职工带薪年休假实施办法

**发布部门：** [人力资源和社会保障部](https://www.pkulaw.com/law/chl?Aggs.IssueDepartment=60236) [机构沿革](javascript:void(0))

**发文字号：**中华人民共和国人力资源和社会保障部令第1号

**发布日期：**2008.09.18

**实施日期：**2008.09.18

**时效性：** [现行有效](https://www.pkulaw.com/law/chl?Aggs.TimelinessDic=01)

**效力级别：** [部门规章](https://www.pkulaw.com/law/chl?Aggs.EffectivenessDic=XE0301)

**法规类别：** [人事综合规定](https://www.pkulaw.com/law/chl?Aggs.Category=08801) [疫情防控](https://www.pkulaw.com/law/chl?Aggs.Category=122)

中华人民共和国人力资源和社会保障部令
(第1号)

　　《企业职工带薪年休假实施办法》已于2008年7月17日经人力资源和社会保障部第6次部务会议通过，现予公布，自公布之日起施行。

部长 尹蔚民
2008年9月18日

　　企业职工带薪年休假实施办法

　　**第一条**　为了实施《[职工带薪年休假条例](https://www.pkulaw.com/chl/da3d4a4e60aa59d2bdfb.html)》（以下简称[条例](https://www.pkulaw.com/chl/da3d4a4e60aa59d2bdfb.html)），制定本实施办法。

　　**第二条**　中华人民共和国境内的企业、民办非企业单位、有雇工的个体工商户等单位（以下称用人单位）和与其建立劳动关系的职工，适用本办法。

　　**第三条**　职工连续工作满12个月以上的，享受带薪年休假（以下简称年休假）。

　　**第四条**　年休假天数根据职工累计工作时间确定。职工在同一或者不同用人单位工作期间，以及依照法律、行政法规或者国务院规定视同工作期间，应当计为累计工作时间。

　　**第五条**　职工新进用人单位且符合本办法第三条规定的，当年度年休假天数，按照在本单位剩余日历天数折算确定，折算后不足1整天的部分不享受年休假。

　　前款规定的折算方法为： （当年度在本单位剩余日历天数÷365天）×职工本人全年应当享受的年休假天数。

　　**第六条**　职工依法享受的探亲假、婚丧假、产假等国家规定的假期以及因工伤停工留薪期间不计入年休假假期。

　　**第七条**　职工享受寒暑假天数多于其年休假天数的，不享受当年的年休假。确因工作需要，职工享受的寒暑假天数少于其年休假天数的，用人单位应当安排补足年休假天数。

　　**第八条**　职工已享受当年的年休假，年度内又出现[条例](https://www.pkulaw.com/chl/da3d4a4e60aa59d2bdfb.html)第[四条](https://www.pkulaw.com/chl/da3d4a4e60aa59d2bdfb.html#tiao_4)第（[二](https://www.pkulaw.com/chl/da3d4a4e60aa59d2bdfb.html#tiao_4_kuan_1_xiang_2)）、（[三](https://www.pkulaw.com/chl/da3d4a4e60aa59d2bdfb.html#tiao_4_kuan_1_xiang_3)）、（[四](https://www.pkulaw.com/chl/da3d4a4e60aa59d2bdfb.html#tiao_4_kuan_1_xiang_4)）、[（五）项](https://www.pkulaw.com/chl/da3d4a4e60aa59d2bdfb.html#tiao_4_kuan_1_xiang_5)规定情形之一的，不享受下一年度的年休假。

　　**第九条**　用人单位根据生产、工作的具体情况，并考虑职工本人意愿，统筹安排年休假。用人单位确因工作需要不能安排职工年休假或者跨1个年度安排年休假的，应征得职工本人同意。

　　**第十条**　用人单位经职工同意不安排年休假或者安排职工年休假天数少于应休年休假天数，应当在本年度内对职工应休未休年休假天数，按照其日工资收入的300%支付未休年休假工资报酬，其中包含用人单位支付职工正常工作期间的工资收入。

　　用人单位安排职工休年休假，但是职工因本人原因且书面提出不休年休假的，用人单位可以只支付其正常工作期间的工资收入。

　　**第十一条**　计算未休年休假工资报酬的日工资收入按照职工本人的月工资除以月计薪天数（21.75天）进行折算。

　　前款所称月工资是指职工在用人单位支付其未休年休假工资报酬前12个月剔除加班工资后的月平均工资。在本用人单位工作时间不满12个月的，按实际月份计算月平均工资。

　　职工在年休假期间享受与正常工作期间相同的工资收入。实行计件工资、提成工资或者其他绩效工资制的职工，日工资收入的计发办法按照本条第一款、第二款的规定执行。

　　**第十二条**　用人单位与职工解除或者终止劳动合同时，当年度未安排职工休满应休年休假的，应当按照职工当年已工作时间折算应休未休年休假天数并支付未休年休假工资报酬，但折算后不足1整天的部分不支付未休年休假工资报酬。

　　前款规定的折算方法为： （当年度在本单位已过日历天数÷365天）×职工本人全年应当享受的年休假天数-当年度已安排年休假天数。

　　用人单位当年已安排职工年休假的，多于折算应休年休假的天数不再扣回。

　　**第十三条**　劳动合同、集体合同约定的或者用人单位规章制度规定的年休假天数、未休年休假工资报酬高于法定标准的，用人单位应当按照有关约定或者规定执行。

　　**第十四条**　劳务派遣单位的职工符合本办法第三条规定条件的，享受年休假。

　　被派遣职工在劳动合同期限内无工作期间由劳务派遣单位依法支付劳动报酬的天数多于其全年应当享受的年休假天数的，不享受当年的年休假；少于其全年应当享受的年休假天数的，劳务派遣单位、用工单位应当协商安排补足被派遣职工年休假天数。

　　**第十五条**　县级以上地方人民政府劳动行政部门应当依法监督检查用人单位执行[条例](https://www.pkulaw.com/chl/da3d4a4e60aa59d2bdfb.html)及本办法的情况。

　　用人单位不安排职工休年休假又不依照[条例](https://www.pkulaw.com/chl/da3d4a4e60aa59d2bdfb.html)及本办法规定支付未休年休假工资报酬的，由县级以上地方人民政府劳动行政部门依据职权责令限期改正；对逾期不改正的，除责令该用人单位支付未休年休假工资报酬外，用人单位还应当按照未休年休假工资报酬的数额向职工加付赔偿金；对拒不执行支付未休年休假工资报酬、赔偿金行政处理决定的，由劳动行政部门申请人民法院强制执行。

　　**第十六条**　职工与用人单位因年休假发生劳动争议的，依照劳动争议处理的规定处理。

　　**第十七条**　除法律、行政法规或者国务院另有规定外，机关、事业单位、社会团体和与其建立劳动关系的职工，依照本办法执行。

　　船员的年休假按 《[中华人民共和国船员条例](https://www.pkulaw.com/chl/4d75aecbb3e59685bdfb.html)》执行。

　　**第十八条**　本办法中的“年度”是指公历年度。

　　**第十九条**　本办法自发布之日起施行。

© 北大法宝：（[www.pkulaw.com](https://www.pkulaw.com/)) 专业提供法律信息、法学知识和法律软件领域各类解决方案。北大法宝为您提供丰富的参考资料，正式引用法规条文时请与标准文本核对。
欢迎查看所有[产品和服务](https://www.pkulaw.com/helps/2.html)。法宝快讯：[如何快速找到您需要的检索结果？](https://www.pkulaw.com/helps/70.html)[法宝](https://www.pkulaw.com/helps/69.html)V6有何新特色？

扫描二维码阅读原文

**原文链接：**<https://www.pkulaw.com/chl/23d57db4989624b1bdfb.html>
